# Combined ultrasound–hydrothermal pretreatment enhances moisture diffusivity, energy efficiency, and quality retention during hot air drying of holy basil (*Ocimum sanctum* L.)

**DOI:** 10.1016/j.ultsonch.2026.107865

**Published:** 2026-04-20

**Authors:** Suluh Pambudi, Duanghathai Khamon, Tai Van Ngo, Wanphut Saechua, Jiraporn Sripinyowanich Jongyingcharoen

**Affiliations:** a Department of Biosystems and Agricultural Engineering, School of Engineering, King Mongkut’s Institute of Technology Ladkrabang, Ladkrabang, Bangkok 10520, Thailand; b Department of Food Technology, School of Food Industry, King Mongkut’s Institute of Technology Ladkrabang, Ladkrabang, Bangkok 10520, Thailand

**Keywords:** Ultrasonic pretreatment, Hydrothermal pretreatment, Pretreatment combination, Energy-efficient dehydration, Quality retention

## Abstract

Drying of holy basil (*Ocimum sanctum* L.) is limited by slow internal moisture diffusion and severe degradation of heat-sensitive pigments and bioactive compounds during prolonged thermal exposure. This study investigates the combined effects of ultrasonic and hydrothermal pretreatments on moisture transport, energy efficiency, and quality retention during hot air drying at 60 °C. Ultrasonication (30 and 60 min), hot-water blanching, steam blanching, and their combinations were systematically evaluated through drying kinetics, effective moisture diffusivity (D_eff_), microstructural analysis, POD enzyme activity, specific energy consumption (SEC), and comprehensive quality assessment. Drying occurred entirely in the falling-rate regime, confirming diffusion-controlled moisture transport. The combined ultrasound–blanching treatment (US30B) exhibited the greatest changes in drying performance, enlarging stomatal openings and enhancing moisture transport during drying, which increased D_eff_ nearly threefold (from 1.70 × 10^−11^ to 5.02 × 10^−11^ m^2^/s) and reduced drying time from 100 to 30 min. This transport intensification decreased total SEC by 11% while simultaneously minimizing shrinkage and enhancing rehydration capacity. Importantly, accelerated moisture removal may have reduced thermal and oxidative degradation, resulting in superior retention of chlorophyll, phenolics, flavonoids, and antioxidant activity (p ≤ 0.05), alongside improved green color stability. These findings suggest that pretreatment strategies that enhance internal moisture transport may improve both energy efficiency and functional quality in heat-sensitive leafy herbs. The combined ultrasound–hydrothermal pretreatment shows potential as a practical approach for improving drying performance and quality retention in herbal dehydration.

## Graphical abstract

**Figure:**
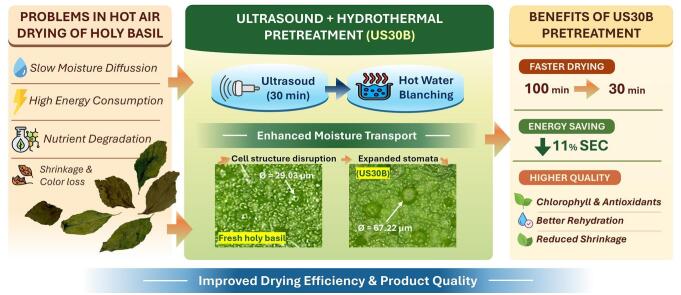

## 1 Introduction

The drying process is particularly critical for medicinal herbs such as holy basil (*Ocimum sanctum* L.), as it directly determines the preservation of bioactive compounds including phenolic acids, flavonoids, chlorophyll, and essential oils. These constituents are responsible for the herb’s antioxidant, anti-inflammatory, and antimicrobial activities and thus define its therapeutic and commercial value [1], [2], [3]. Drying must therefore achieve a delicate balance: sufficient moisture removal to ensure microbial stability while minimizing thermal and enzymatic degradation of heat-sensitive compounds. Inadequate control of drying conditions can accelerate pigment loss, structural collapse, and enzymatic browning, leading to diminished quality and reduced consumer acceptance in food and pharmaceutical applications [2], [4].

Previous studies have demonstrated that both drying temperature and method strongly influence the retention of bioactive compounds. Heat-intensive processes can induce substantial losses of chlorophyll, phenolics, and flavonoids, although the magnitude and direction of these changes depend on operating conditions [5], [6]. While certain techniques, such as moderate-temperature microwave or oven drying, have shown improved phenolic retention under optimized conditions, excessive thermal or mechanical stress often results in severe degradation of volatile compounds and essential oils due to cell wall rupture and enhanced leaching [7], [8]. These findings highlight the need for drying strategies that limit thermal exposure while maintaining efficient moisture removal.

Despite the availability of advanced drying technologies, hot air (HA) drying remains the most widely applied method in industrial herb processing due to its simplicity, low capital cost, and scalability. Notably, HA drying has in several cases demonstrated comparable or even superior retention of total phenolic content (TPC), total flavonoid content (TFC), and antioxidant activity compared with more energy-intensive techniques, provided that excessive drying times can be avoided [9], [10], [11]. However, the primary limitation of HA drying lies in its low moisture diffusion efficiency, which prolongs drying duration, intensifies thermal exposure, and increases energy consumption. Consequently, improving internal moisture transport rather than replacing the drying method itself represents a more practical pathway toward energy-efficient processing.

In this context, pretreatment has emerged as a key strategy to overcome internal and surface mass-transfer resistance prior to drying. Ultrasonic pretreatment has attracted considerable interest due to its ability to generate acoustic cavitation, inducing microstructural disruption, pore formation, and enhanced internal moisture diffusivity [12], [13], [14]. In contrast, hydrothermal pretreatments such as blanching and steaming have been reported to inactivate degradative enzymes such as peroxidase and chlorophyllase that contribute to pigment and quality loss during drying [15], [16]. Importantly, these two pretreatment approaches target fundamentally different transport and degradation mechanisms: ultrasound has been reported to enhance internal moisture transport, whereas hydrothermal treatment may reduce external mass transfer resistance and biochemical deterioration.

However, existing studies on holy basil drying have largely examined ultrasonic or hydrothermal pretreatments independently, with limited attention to their combination effects. More critically, the interaction between structural alteration and drying behavior, POD enzyme inactivation, moisture diffusion behavior, and energy consumption during HA drying remains poorly understood. Addressing this gap is essential for establishing a rational basis for pretreatment-assisted drying design. Therefore, this study aims to elucidate the combination role of ultrasound–hydrothermal pretreatment in enhancing hot air drying of holy basil leaves. By integrating drying kinetics, effective moisture diffusivity, microstructural observation, POD enzyme activity, product quality, and specific energy consumption analyses, this work provides insight into how pretreatment may influence moisture transport behavior during drying. The findings contribute to the development of energy-efficient drying strategies for heat-sensitive leafy medicinal herbs.

## 2 Materials and methods

### 2.1 Sample preparation

Fresh holy basil leaves (*Ocimum sanctum* L*.*) were used in this study, purchased from a Makro supermarket (Thailand) and stored at room temperature (25 °C) prior to experimental use. The leaves were thoroughly washed with water and size-selected to ensure uniformity for the experiments. The detailed properties of the leaves are summarized in Table 1.

**Table 1:** Characteristics of fresh holy basil leaves used in the experiments.

Property	Value
Width (cm)	2.61 ± 0.01
Length (cm)	5.12 ± 0.01
Surface area (cm^2^)	15.26 ± 0.32
Initial moisture content (g_w_/g_dm_)	0.89 ± 0.01
L* (lightness)	35.66 ± 0.23
a* (red/green)	−9.45 ± 0.19
b* (yellow/blue)	21.02 ± 0.41

### 2.2 Experimental design

The experimental procedures of this study were divided into two main objectives. The first objective focused on investigating changes in the properties of holy basil leaves during ultrasonic and hydrothermal pretreatments. The quality attributes evaluated during pretreatment included colorimetric, peroxidase enzyme activity, and leaf stomatal structure. Peroxidase activity was assessed to analyze enzyme inhibition, while leaf stomata was examined under a stereoscopic light microscope to observe structural changes caused by pretreatment. Pretreatment served as a preparation step prior to HA drying, aiming to accelerate the drying process and improve product quality. Five pretreatment methods were applied: ultrasonic pretreatment for 30  min (US30) and 60  min (US60), ultrasonic pretreatment for 30  min combined with hot water blanching (US30B), hot water blanching (B), and steam blanching (SB). The unpretreated sample served as the control (C).

**Fig. 1:**
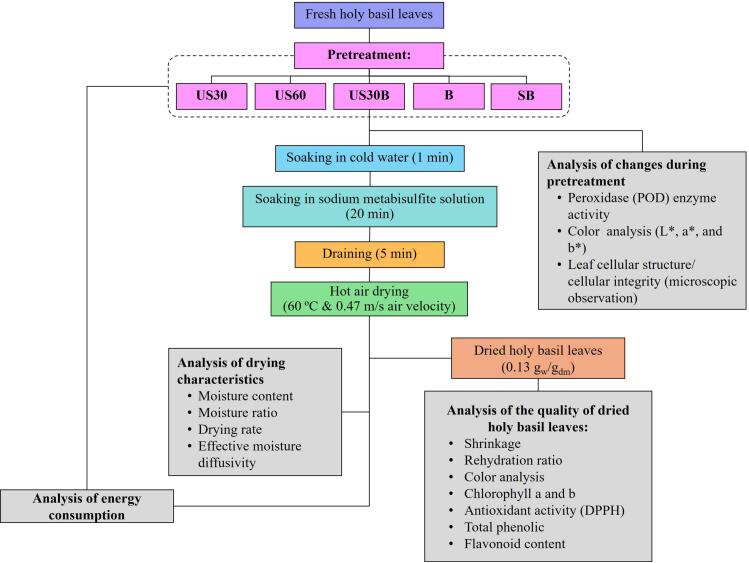
Schematic diagram of experimental design and analytical workflow.

The second objective focused on evaluating the influence of ultrasonic and hydrothermal pretreatments on HA drying behavior and the quality of dried holy basil leaves. Moisture ratio and drying rate were analyzed using drying models, followed by an evaluation of effective moisture diffusivity. In addition, the color and total color change (ΔE*) of the dried samples were measured. Physical quality attributes, such as shrinkage percentage and rehydration ratio, were assessed along with biochemical properties, including chlorophyll *a* and b content. Moreover, selected pretreatment methods were further evaluated for their effects on antioxidant activity (DPPH), TPC, and TFC. A schematic diagram outlining the experimental design and subsequent analyses is presented in Fig. 1.

### 2.3 Ultrasonic and hydrothermal pretreatment procedures

For each experiment, 15  g of size-selected holy basil leaves were used for pretreatment. Ultrasonic pretreatment was conducted using a high-power ultrasonic water bath (DT 510H, Bandelin electronic GmbH&Co.KG, Germany) having 2 L of ambient temperature water at a frequency of 20  kHz. The ultrasonic system operates at a fixed power without user-adjustable settings. Therefore, the actual energy input was quantified using an energy meter for the purpose of specific energy consumption (SEC) calculation. The leaves were subjected to ultrasonic treatment for either 30  min (US30) or 60  min (US60). For combined ultrasonic and hot water blanching pretreatment (US30B), leaves were first treated ultrasonically under the same conditions for 30  min, followed by blanching in 1  L of boiling water at 97 °C for 90  s using a water bath (WB 14, Memmert GmbH + Co. KG, Germany). Hot water blanching alone (B) was performed by immersing the leaves in 1  L of boiling water at 97 °C for 90  s. Steam blanching (SB) involved placing leaves in a thin layer on a perforated tray and steaming for 90  s using an electric steamer (VC-100665, Tefal, Groupe SEB, France). After pretreatments, leaves were immediately cooled in cold water for 60  s, then soaked in a 0.2% (w/v) sodium metabisulfite solution as an antimicrobial agent for 20  min. The leaves were left to drain for 5 min to remove surface moisture before being subjected to HA drying.

### 2.4 Hot-air drying procedure and drying behavior analysis

Following pretreatment, the holy basil leaves were dried using an HA oven (UF160, Memmert, Memmert GmbH + Co.KG, Schwabach, Germany) at a constant temperature of 60 °C with an air velocity of 0.47  m/s for 2 hr. Approximately 15  g of pretreated leaves were spread in a single layer on perforated trays to ensure uniform drying. The sample weight was recorded at predetermined time intervals (5, 10, 15, 30, 45, 60, 80, and 100 min) using a digital balance with an accuracy of ± 0.0001  g. Sampling at these intervals was continued only until the moisture content of the leaves reached 0.13 g_w_/g_dm_, which was defined as the target final moisture content for this study.

For the determination of moisture content, 1  g of pretreated holy basil leaves were placed in 2.5  oz moisture can previously dried to remove residual moisture. Two replicates per treatment were used. The samples were dried in an HA oven at 105 ± 2 °C for 24  h. The moisture content of the leaves was calculated to get the dry matter using Eq. (1).

(1) $$M=\frac{W_{w}-W_{d}}{W_{d}}$$

where M is the moisture content (g_w_/g_dm_), W_w_ is the wet weight of the material (g), and W_d_ is the dry matter weight of the material (g). The moisture ratio during the drying process was analyzed as a function of drying time based on Eq. (2).

(2) $$MR=\frac{M_{t}-\;M_{e}}{M_{i}-\;M_{e}}$$

where MR is the moisture ratio, M_t_ is the moisture content at specific drying time, M_i_ is the initial moisture content, and M_e_ is the equilibrium moisture content. In this study, the equilibrium moisture content was assumed negligible under the applied thin-layer drying conditions, consistent with previous studies on leafy materials [17]. Furthermore, the drying rate was calculated according to Eq. (3).

(3) $$DR=\frac{M_{t}-\;M_{t+dt}}{dt}$$

where DR is the drying rate (g_w_/g_dm_·min), M_t+dt_ is moisture content at the t + dt, and t is drying time. This approach allowed quantification of the water evaporated per unit time during the HA drying process.

### 2.5 Estimation of effective moisture diffusivity

The effective moisture diffusivity of holy basil leaves during HA drying was determined using Fick’s second law of diffusion, assuming moisture movement occurred primarily in one dimension, and the leaves had a uniform initial moisture distribution. For thin layer drying, the solution to Fick’s second law for a slab geometry is expressed in a simplified form by considering only the first term of the series, which is valid for longer drying times, as in Eq. (4).

(4) $$MR=\frac{8}{\pi^{2}}exp\;\left(\frac{\pi^{2}D_{eff}t}{{4L}^{2}}\right)$$

where D_eff_ is the effective moisture diffusivity (m^2^/s), t is the drying time (s), and L is the half-thickness of the leaf (m). A linear regression of lnMR versus drying time was used to determine the slope, from which D_eff_ was calculated.

### 2.6 Specific energy consumption analysis

The specific energy consumption (SEC) was analyzed to evaluate the energy efficiency and cost-effectiveness of each pretreatment during dried holy basil production. Energy consumption measurements were conducted using a METREL MI 2883 energy meter, which was connected to the electrical panel supplying the experimental setup. A single-phase voltage line and a neutral wire were connected while performing both the pretreatment and HA drying processes. SEC reflects the efficiency of energy usage and provides insight into the energy cost associated with industrial production. It was defined as the amount of energy required to remove 1 kg of water from the material and is therefore expressed in kWh/kg, as shown in Eq. (5).

(5) $$SEC=\frac{E_{t}}{W_{0}}$$

where E_t_ represents the total energy required (kWh), and W_0_ denotes the mass of water removed (kg).

### 2.7 Quality analysis of fresh, pretreated, and dried holy basil

#### 2.7.1 Determination of peroxidase enzyme activity

Peroxidase enzyme activity in the leaf samples was determined using a qualitative guaiacol oxidation assay. Leaf samples were homogenized using a Philips HR2058 vegetable blender at 280 W, with 30 mL of distilled water added per sample. The resulting homogenate was filtered through Whatman No. 1 filter paper to obtain the enzyme extract. For the assay, two 2 mL aliquots of the filtered enzyme extract were transferred into separate test tubes, each subsequently diluted with 20 mL of distilled water. To one tube (experimental sample), 1 mL of 0.5% (v/v) guaiacol and 1 mL of 0.08% (v/v) hydrogen peroxide were added. The mixture was thoroughly inverted to ensure complete reagent distribution. The reaction was timed immediately upon reagent addition, and the development of a distinct brown color within 3 min 50 s was indicative of positive peroxidase activity. Conversely, the absence of any color change within this observation period was considered negative for peroxidase activity. A control sample was prepared by combining a 2 mL aliquot of the enzyme extract with 20 mL of distilled water, omitting the guaiacol addition, to serve as a visual reference for the inherent color of the extract. The assay provides a qualitative indication of residual POD activity rather than a quantitative measure of enzyme kinetics.

#### 2.7.2 Determination of color and color difference

For color analysis, a 5 g sample was homogenized using a Philips HR2058 vegetable blender operating at 280 W for 2 min. The color attributes of the resulting homogenized sample were quantitatively assessed using a Spectrocolorimeter (ColorFlex, version 1.72, Hunter Associates Laboratory, Inc., USA). This instrument was utilized to measure the L*, a*, and b* color space values, representing brightness (0–100; black to white), redness (– to +; green to red), and yellowness (– to +; blue to yellow), respectively. Sample aliquots were placed in quartz cuvettes, and all measurements were performed triplicate. The total color difference (ΔE*) was subsequently calculated to evaluate the overall color changes as compared to fresh leaves. The calculation followed the formula presented in Eq. (6).

(6) $${\Delta E}^{*}=\sqrt{{\left({L_{0}}^{*}-\;L^{*}\right)}^{2}+{\left({a_{0}}^{*}-\;a^{*}\right)}^{2}+{\left({b_{0}}^{*}-\;b^{*}\right)}^{2}}$$

#### 2.7.3 Examination of cellular structure

The stomatal structure of pretreated leaf samples was examined using a 3D microscope (ZEISS Stemi 508) at 500 × magnification. Prior to observation, leaves were gently blotted to remove any excess surface water. This microscopic examination allowed for the visualization and assessment of the three-dimensional cellular and tissue structural changes induced by the different pretreatment methods.

#### 2.7.4 Shrinkage percentage

Shrinkage percentage was determined to quantify the reduction in leaf area during drying. Leaf area was calculated using ImageJ software, and shrinkage percentage was computed according to Eq. (7).

(7) $$Shrinkage\;\left(\%\right)=\frac{A_{fresh}-A_{dried}}{A_{fresh}}\times 100$$

where A_fresh_ and A_dried_ correspond to the leaf area of fresh and dried holy basil, respectively.

#### 2.7.5 Rehydration ratio

The rehydration ratio was determined by immersing ten randomly selected dried leaves from each pretreatment group into a water bath (DT 510H, Bandelin electronic GmbH & Co.KG, Germany) maintained at 30 °C. At 5-min intervals, for a total duration of 35 min, leaves were carefully removed from the water bath. Excess surface water was gently blotted, and their weight was recorded using an analytical balance. The rehydration ratio was calculated as shown in Eq. (8).

(8) $$RR=\frac{m_{rehydrated}}{m_{dried}}$$

where m_dried_ and m_rehydrated_ are the weights of the sample before (dried leaves) and after rehydration (g), respectively.

#### 2.7.6 Determination of chlorophyll *a* and b content

Leaf samples (1 g) were finely ground while avoiding major veins and extracted with 20 mL of acetone for 20 min. The homogenate was filtered to remove particulate matter, and the filtrate volume was adjusted to 30 mL with acetone. To prevent pigment degradation, all extracts were protected from light by wrapping the containers in aluminum foil. Absorbance was recorded at 663 nm and 645 nm using a spectrophotometer (Genesys 10S UV–VIS). Chlorophyll *a* and chlorophyll *b* contents were calculated according to Eqs. (9), (10) according to Arnon [18] method.

(9) $$Chlorophyll\;a\;=\;\left[12.7\left(OD663\right)-2.69\left(OD645\right)\right]\times \frac{V}{1000\times m}$$

(10) $$Chlorophyll\;b\;=\;\left[22.9\left(OD645\right)-4.68\left(OD663\right)\right]\;\times \frac{V}{1000\times m}$$

where V is the final volume of the chlorophyll extract (mL), m is the sample mass (g), and OD represents the absorbance at the respective wavelengths.

#### 2.7.7 Determination of total phenolic content

Total phenolic content was quantified using a modified Folin–Ciocalteu colorimetric method [19]. A 125 µL aliquot of the sample extract was mixed with 500 µL of distilled water and 125 µL of Folin reagent, and the mixture was allowed to react for 6 min. Subsequently, 1,250 µL of 7% sodium carbonate and 1,000 µL of distilled water were added. The reaction mixture was incubated for 90 min at room temperature, protected from light. Absorbance was then measured at 760 nm using a spectrophotometer. All analyses were conducted in triplicate. Total phenolic content was determined using a gallic acid calibration curve (20–200 µg/L) and expressed as mg gallic acid equivalents per gram of sample (mg GAE/g).

#### 2.7.8 Determination of total flavonoid content

Total flavonoid content was measured following the method of Wolfe, et al. [19]. A 250 µL aliquot of the sample extract was mixed with 1,250 µL of distilled water and 75 µL of 5% sodium nitrite solution, and the mixture was allowed to stand for 5 min. Subsequently, 150 µL of 10% aluminum chloride and 500 µL of 1 M sodium hydroxide were added, followed by 275 µL of distilled water. After a 6 min reaction period, absorbance was recorded at 510 nm using a spectrophotometer. All measurements were conducted in triplicate. Total flavonoid content was quantified using a catechin calibration curve and expressed as mg catechin equivalents per gram of sample (mg CE/g).

#### 2.7.9 Determination of antioxidant activity

The antioxidant activity was quantified using the 2,2-diphenyl-1-picrylhydrazyl (DPPH) radical scavenging assay following Zhu, et al. [20]. A 0.1 mM DPPH solution was prepared in 95% ethanol, and 1 mL of this solution was mixed with 1 mL of basil extract. The mixture was incubated for 30 min in the dark to prevent photodegradation. Absorbance was recorded at 517 nm using a spectrophotometer. All measurements were performed in triplicate. Trolox (1–10 µg/mL) served as the standard for generating a calibration curve, and antioxidant activity was expressed as mg Trolox equivalents per gram of sample (mg TE/g).

### 2.8 Statistical and multivariate analysis

To evaluate the changes in the quality of holy basil leaves during ultrasonic and hydrothermal pretreatments, all experimental data were analyzed using SPSS software. Data were subjected to one-way analysis of variance (ANOVA), and mean differences were compared using the Least Significant Difference (LSD) test at a 95% confidence level. Principal component analysis (PCA) was employed to examine the multivariate relationships among variables, as well as to visualize the clustering patterns between treatments. Pearson correlation analysis was used to determine the strength and direction of linear relationships among the measured variables.

## 3 Results and discussion

### 3.1 Effects of pretreatment on enzymatic activity, microstructure, and initial color attributes

The qualitative evaluation of peroxidase enzyme activity in holy basil leaves subjected to various ultrasonic and hydrothermal pretreatments are presented in Table 2. Peroxidase serves as a useful indicator for evaluating the effectiveness of thermal pretreatments due to its relatively high thermostability in plant tissues [21]. Residual peroxidase activity has been associated with enzymatic degradation and color deterioration in plant-based food products during processing and storage [22]. In the US30 and US60, strong peroxidase activity (+++) persisted during the first 15 min. A gradual decline in color intensity (to ++) was observed after 30 min and remained relatively stable up to 60 min, suggesting partial denaturation of the enzyme rather than full deactivation. This observation is consistent with previous findings on ultrasonic treatment of mulberry (*Morus nigra*) juice, where peroxidase exhibited notable thermal and structural stability under sonication alone. Such results confirm that peroxidase is relatively resistant to inactivation by ultrasound treatment, likely due to its compact tertiary structure and high thermostability compared to other oxidoreductases such as polyphenol oxidase (PPO) activity [23].

**Table 2:** Peroxidase enzyme activity of holy basil leaves under different pretreatment processes.

Treatment methods	Ultrasonic pretreatment				Hydrothermal pretreatment		
	Pretreatment time (min)				Pretreatment time (s)		
	5	15	30	60	30	90	180
Fresh leaf							
US30							
US60							
US30B							
B							
SB							

“+” indicates the development of reddish-brown coloration in the test, confirming the presence of peroxidase enzyme activity. A greater number of “+” signs correspond to higher color intensity. “-” indicates no reddish-brown coloration, confirming the absence of peroxidase enzyme activity.

In contrast, hydrothermal pretreatments (B and SB) resulted in no detectable peroxidase activity under the qualitative assay conditions, likely due to thermal denaturation. In the US30B treatment, despite exhibiting lower reddish-brown discoloration after ultrasonication, no peroxidase activity was detected within 90 s, comparable to B and SB. The higher efficiency of hydrothermal treatments results from rapid enzyme inactivation, which is strongly influenced by blanching temperature and time [24]. Similarly, in the case of sweet corn kernels, a 95% reduction in peroxidase activity was achieved after 90 s of hot water blanching at 90 °C, indicating that comparable thermal conditions are required for effective enzyme inactivation [25]. High-humidity hot air impingement blanching, which shares principles with superheated steam, has similarly demonstrated efficient inactivation of both peroxidase and polyphenol oxidase within short durations, with no activity detected after 120 s [26].

The variation in colorimetric parameters among pretreatments reflects differences in chlorophyll pigment retention and enzyme inactivation intensity (Table 3). Generally, B and SB exhibited more positive a* values compared to US30 and US60. Furthermore, the combined pretreatment (US30B) demonstrated intermediate color characteristics, differing significantly from both ultrasound-only and hydrothermal-only pretreatments (p < 0.05). It exhibited the highest L* value, the lowest a* value, and a significantly lower b* value compared with the other pretreatments. Overall color difference (ΔE) values further confirmed the pretreatment-dependent variation in color preservation, with US30B and SB exhibiting the lowest ΔE, indicating closer visual similarity to fresh leaves. In contrast, US30, US60, and B showed significantly higher ΔE values (p ≤ 0.05), reflecting greater cumulative color deviation arising from either mechanical stress or thermal pigment degradation. Such a phenomenon occurs because blanching and steam blanching preserve color by inactivating browning enzymes, whereas ultrasonication disrupts cells without deactivating these enzymes, leading to instant enzymatic browning within minutes prior to drying. Similar quality trade-offs have been reported for water blanching of galega kale and for ultrasonic pretreatment applied to parsley leaves [15], [27]. Furthermore, b* values were significantly elevated (p < 0.05) in all pretreated samples relative to fresh leaves, indicating partial chlorophyll degradation and a relative increase in thermally stable carotenoids.

**Table 3:** Colorimetric analysis of holy basil leaves under various pretreatment conditions.

Pretreatment	Time (min)	L*	a*	b*	ΔE
Fresh leaf	–	35.78 ± 0.09^c^	–9.45 ± 0.10^c^	21.02 ± 0.09^e^	−
US30	30	36.47 ± 0.06^b^	–12.42 ± 0.06^b^	28.83 ± 0.04^a^	8.38 ± 0.03^a^
US60	60	37.29 ± 0.12^a^	–12.84 ± 0.04^a^	27.43 ± 0.05^c^	7.41 ± 0.00^b^
US30B	31.5	37.52 ± 0.12^a^	–7.65 ± 0.02^f^	27.15 ± 0.03^d^	6.62 ± 0.07^c^
B	1.5	33.88 ± 0.13^d^	–7.95 ± 0.04^e^	27.98 ± 0.03^b^	7.37 ± 0.08^b^
SB	1.5	37.38 ± 0.12^a^	–8.15 ± 0.05^d^	27.35 ± 0.03^c^	6.66 ± 0.06^c^

Different letters in each column indicate significant differences at p ≤ 0.05.

**Fig. 2:**
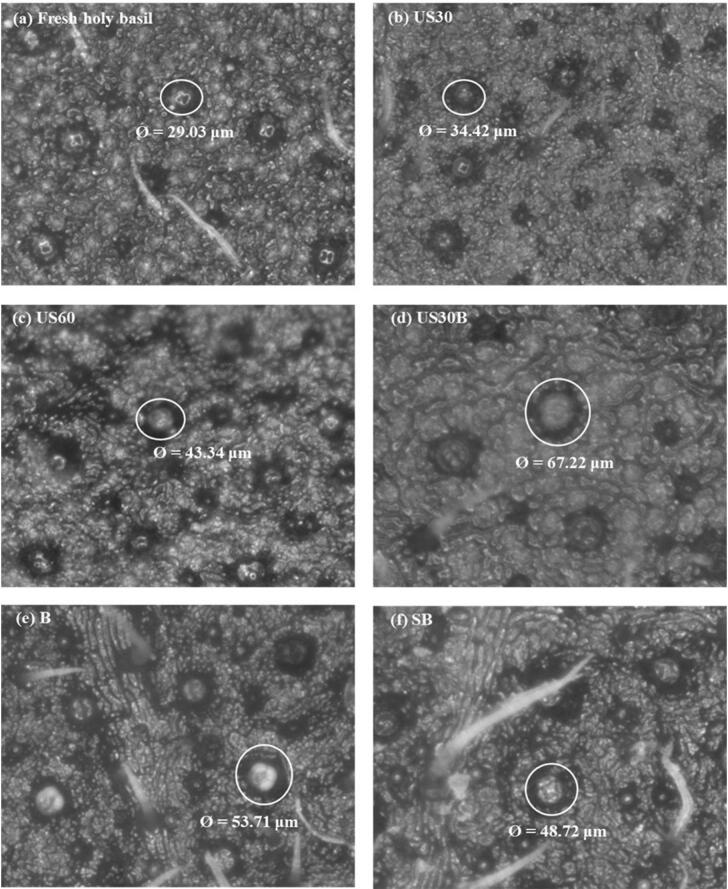
Microscopic analysis of stomatal and cellular structural changes in holy basil leaves under pretreatments.

These phenomena were further evidenced by microscopic analysis of fresh and pretreated leaves. As shown in Fig. 2, untreated holy basil leaves exhibited compact epidermal tissue. The relatively small stomatal openings (29.03 µm) suggest high resistance to internal moisture transport. US30 and US60 induced pronounced microstructural disruption, characterized by enlarged stomatal apertures (34.42–43.34 µm) and partial loosening of surrounding tissues, which can be attributed to cavitation and microstreaming effects. In contrast, B and SB produced more pronounced stomatal enlargement (48.72–53.71 µm), reflecting predominantly thermal rather than mechanical effects. Notably, the combined US30B pretreatment resulted in the greatest observable changes in stomatal opening (67.22 µm) and more homogenized tissue morphology. This is consistent with the observed enhancement in moisture transport. Furthermore, this phenomenon was similar to the pretreatment using pulsed electric field on Genovese basil. In that research, it was found that pulsed electric field treatments could induce guard cell electroporation, leading to sustained stomatal opening, which in turn resulted in significantly higher moisture diffusivity and faster drying kinetics [28].

### 3.2 Influence of pretreatment methods on drying characteristics

**Fig. 3:**
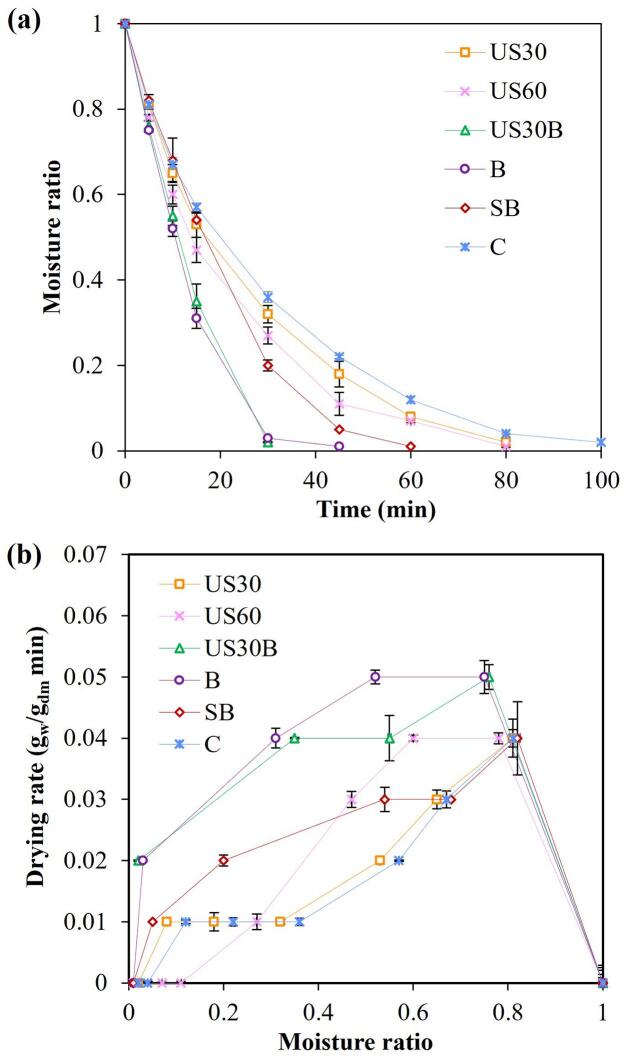
Drying characteristics of holy basil leaves under different pretreatment methods: (a) moisture ratio vs. drying time, and (b) drying rate vs. moisture ratio.

As shown in Fig. 3a, all samples exhibited a rapid initial moisture reduction during HA drying, followed by a continuously decreasing drying slope. This behavior indicates the absence, or extremely short duration, of a constant-rate period and confirms that drying occurred predominantly in the falling-rate regime. The curvature of the MR–time profiles reflect diffusion-controlled moisture transport, where internal resistance governs drying kinetics rather than surface evaporation [29]. This interpretation is further supported by the DR–MR curves in Fig. 3b, which show no discernible constant DR plateau for any pretreatment. Pretreatment markedly enhanced drying kinetics compared with the control. All pretreated samples reached lower MR more rapidly. This improvement may be associated with pretreatment-induced tissue alteration observed under microscopy (Fig. 2). Enlarged stomatal openings and disrupted cellular structures were associated with enhanced internal moisture migration. Consequently, the control sample exhibited a prolonged drying tail at low MR, reflecting higher diffusion resistance associated with intact cellular structure. Similar diffusion-limited behavior has been reported for thin leafy materials such as parsley and cabbage subjected to HA drying [27], [29].

Among all treatments, the US30B exhibited the highest DR across most of the MR range, resulting in the shortest drying time (30 min), followed B (45 min). Ultrasonic pretreatment alone (US30 and US60) also accelerated drying relative to the control, particularly at intermediate MR (0.3–0.7), indicating enhanced internal moisture diffusivity. However, their DR remained lower than those of treatments incorporating hydrothermal steps, highlighting the additional contribution of surface tissue modification and enzyme inactivation to moisture transport enhancement. At low MR (< 0.2), all treatments exhibited a sharp decline in DR, corresponding to the late falling-rate stage. In this stage, moisture is strongly bound within the solid matrix, and transport is increasingly limited by structural shrinkage, pore collapse, and intensified water–solid interactions. These mechanisms substantially increase diffusion resistance, thereby restricting further moisture removal under HA drying conditions.

### 3.3 Effective moisture diffusivity

**Fig. 4:**
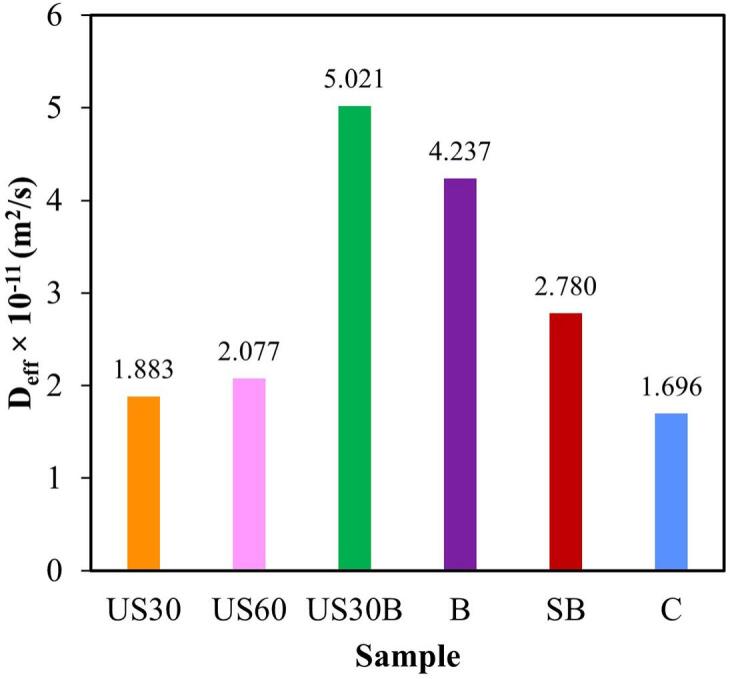
Effective moisture diffusivity (D_eff_) of holy basil leaves subjected to different pretreatment methods during hot air drying.

Effective moisture diffusivity (D_eff_) was used as a physical parameter to quantify internal moisture transport during HA drying. As shown in Fig. 4, pretreatment generally increased D_eff_ relative to control. The highest D_eff_ was achieved by US30B (5.021 × 10^–11^ m^2^/s), followed by B (4.237 × 10^–11^ m^2^/s) and SB (2.780 × 10^–11^ m^2^/s), while US30 and US60 resulted in intermediate diffusivity enhancement. The superior performance of US30B further highlights the improved performance of the combined ultrasound and blanching treatment, which is consistent with the shortest drying time observed for this sample compared with other pretreatments and the control. Furthermore, increasing the ultrasonic durations from 30 to 60 min resulted in a higher D_eff_. This improvement is attributed to the intensified cavitation activity and fragmentation effects at longer treatment, which enhanced moisture turbulence and may have contributed to localized tissue disruption within the holy basil leaf matrix [30]. In addition, the stronger ultrasonic treatment weakened the adhesive and binding forces between the material structure and water molecules, thereby facilitating moisture migration [31].

However, while ultrasonication mainly alters cell wall and tissue structures, blanching was associated with greater enhancement in drying rate when compared with sonication alone. Specifically, when holy basil was pretreated solely by blanching, the magnitude of the D_eff_ was at least twice that achieved by ultrasonication. The moderate increase in D_eff_ observed for ultrasonic pretreatment alone suggests that cavitation primarily enhances internal transport but is insufficient to fully overcome surface resistance of holy basil leaves [29]. Consequently, the textural softening induced by blanching contributed more substantially than ultrasonication to the reduction in drying time. However, the combined pretreatment may facilitate less tortuous moisture movement through the leaf matrix. These distinctions explain the intermediate drying performance observed for individual pretreatments in Section 3.2 and underscore the importance of integrating complementary mechanisms to maximize moisture diffusivity.

### 3.4 Energy consumption and process efficiency

**Fig. 5:**
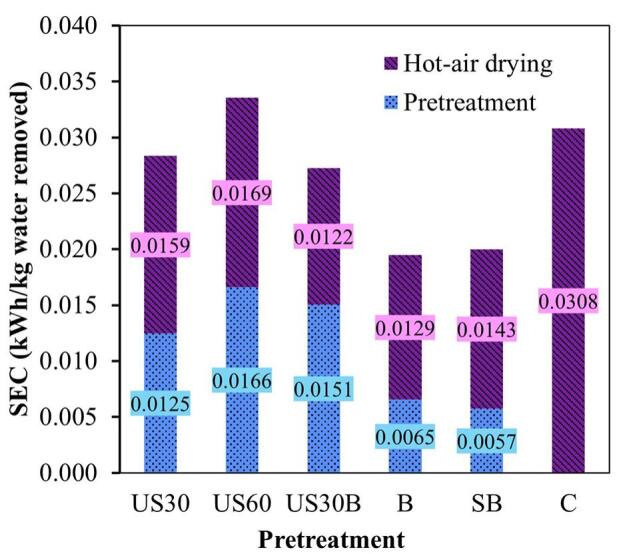
Specific energy consumption in the production of dried holy basil leaves subjected to different pretreatment methods.

Fig. 5 presents the specific energy consumption (SEC) associated with the production of dried holy basil leaves, accounting for both the pretreatment and HA drying stages. The SEC values followed an increasing order of B < SB < US30B < US30 < C < US60, indicating that pretreatments incorporating hydrothermal steps were generally more energy efficient than ultrasound-only treatments. The observed reduction in SEC is associated with improved drying kinetics and reduced drying time, as evidenced by the higher DR and D_eff_ values shown in Figs. 3 and 4. This relationship confirms that improvements in internal mass transfer, which shorten HA drying time, are the primary contributors to reduced overall energy consumption, consistent with previous reports on potato and parsley leaves drying [27], [32].

From an energy-efficiency perspective, the US60 pretreatment should be avoided. Although it reduced SEC during the hot HA stage, its high energy demand during pretreatment resulted in the highest overall SEC, despite achieving a shorter drying time than the control. In general, ultrasonication pretreatments (US30 and US60) exhibited higher SEC during the pretreatment stage compared to hydrothermal methods. Furthermore, they remained less energy efficient than treatments that included a hydrothermal step. Consequently, when energy savings are prioritized, pretreatments involving hydrothermal processes, either alone or combined with short duration of ultrasonication, are recommended as optimal strategies. This trade-off is particularly favorable for industrial HA drying applications, where total energy consumption is dominated by prolonged operating times.

### 3.5 Effect of pretreatment methods on shrinkage of dried holy basil leaves

As summarized in Table 4, US30B exhibited the lowest shrinkage (66.054%), followed by B (70.438%), whereas US30, US60, and SB showed no statistically significant differences among each other (p > 0.05). The highest shrinkage was observed in the control sample (78.690%), which was significantly greater than all pretreated samples, except for US30. This trend is consistent with the leaf projected area results, where US30B and B maintained significantly larger areas (5.183 and 4.513 cm^2^, respectively) compared to the other dried samples. When interpreted alongside drying time (Fig. 3) and D_eff_ (Fig. 4), shorter drying time associated with higher D_eff_ generally coincided with lower shrinkage. This trend reflects the fact that prolonged exposure to elevated temperatures extends the action of capillary stresses and viscoelastic relaxation within leaf tissues, thereby may have contributed to dimensional contraction as dehydration progresses [33]. However, drying time alone did not govern dimensional deformation, as treatments with different drying time (US30 and US60 versus SB) exhibited statistically similar shrinkage values (p > 0.05). This decoupling indicates that, although drying kinetics contribute to shrinkage development, pretreatment-induced tissue modification exerted a more dominant influence on structural deformation than drying duration alone [32].

**Table 4:** Percentage shrinkage of dried holy basil leaves under different pretreatments.

Pretreatment	Leaf area (cm^2^)	Shrinkage percentage (%)
Fresh leaf	15.267 ± 0.31^a^	−
US30	3.615 ± 0.24^de^	76.314 ± 1.66^ab^
US60	3.657 ± 0.10^de^	76.033 ± 1.13^b^
US30B	5.183 ± 0.27^b^	66.054 ± 1.49^d^
B	4.513 ± 0.33^c^	70.438 ± 2.08^c^
SB	3.730 ± 0.20^d^	75.557 ± 0.83^b^
Control	3.250 ± 0.15^e^	78.690 ± 0.58^a^

Different letters in each column indicate significant differences at the 0.05 level (p ≤ 0.05).

In accordance with the present findings, Hawa, et al. [34] reported that hot water blanching promotes pronounced softening of leafy tissues, resulting in a greater extent of pectin gelatinization than steam blanching due to more efficient heat penetration. The formation of a gelatinized pectin network enhances stabilization of the cell wall matrix, thereby limiting excessive structural deformation as the leaves approach equilibrium moisture content during drying. A similar observation was documented by Tabtiang, et al. [35], who reported that banana slices subjected to hot water blanching exhibited reduced shrinkage during hot air drying compared to unpretreated and microwave-treated samples. While ultrasonically pretreated materials have been reported to reach a glassy state more rapidly than untreated controls under identical drying conditions, leading to reduced shrinkage [36], this effect was less pronounced in holy basil under the present conditions. Notably, the combined application of ultrasonication and hot water blanching in US30B led to a greater reduction in shrinkage by facilitating enhanced moisture transport while stabilizing the softened tissue matrix. This interpretation is further supported by the improved color retention observed for US30B, particularly higher L* and lower a* values, which indicate reduced cellular disruption and limited pigment degradation during drying (Table 5).

**Table 5:** Color values of dried holy basil leaves.

Pretreatment	L*	a*	b*	ΔE
US30	34.49 ± 0.11^c^	–3.54 ± 0.13^c^	14.35 ± 0.13^b^	9.06 ± 0.13^a^
US60	34.79 ± 0.13^b^	–3.71 ± 0.11^c^	14.46 ± 0.13^b^	8.83 ± 0.12^b^
US30B	35.25 ± 0.13^a^	–8.43 ± 0.13^a^	13.42 ± 0.16^c^	7.81 ± 0.07^e^
B	31.52 ± 0.13^e^	–8.22 ± 0.15^a^	14.26 ± 0.13^b^	8.14 ± 0.08^d^
SB	35.13 ± 0.16^a^	–7.80 ± 0.11^b^	13.61 ± 0.14^c^	7.74 ± 0.12^e^
Control	32.50 ± 0.11^d^	–2.35 ± 0.13^d^	17.76 ± 0.12^a^	8.43 ± 0.11^c^

Different letters in each column indicate significant differences at p ≤ 0.05.

### 3.6 Influence of pretreatment methods on rehydration behavior of dried holy basil leaves

**Fig. 6:**
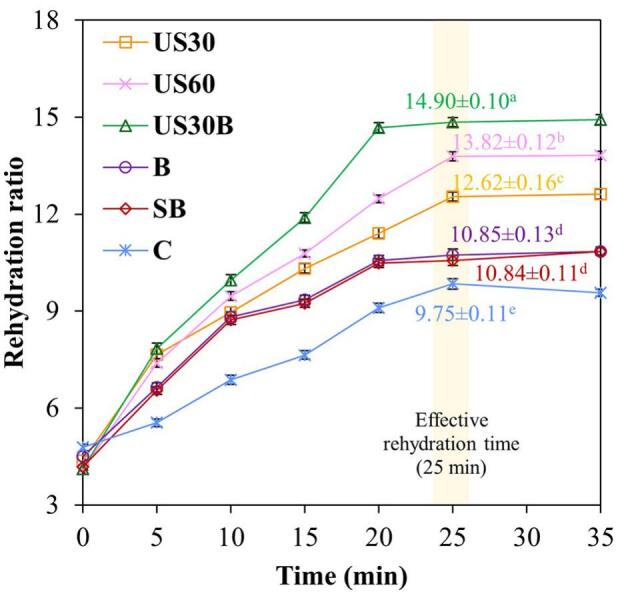
Rehydration ratio of dried holy basil leaves. Different letters in each pretreatment at 25 min of rehydration time indicate significant differences at p ≤ 0.05.

As shown in Fig. 6, pretreatment significantly influenced the rehydration ratio of dried holy basil leaves. Despite the different pretreatments, all samples exhibited the same effective rehydration time of 25 min. However, the rehydration ratios at this time differed significantly (p ≤ 0.05). At this time point, US30B exhibited the highest rehydration ratio (152% vs control), followed by US30 and US60, whereas hydrothermal treatments exhibited significantly lower rehydration ratios. Nevertheless, all pretreated samples generally showed higher rehydration ratios than the control. Similar trends have been reported during the drying of cashew apple, purple-fleshed potatoes, and white cabbage subjected to ultrasonic and hydrothermal pretreatments [29], [37], [38]. The higher rehydration ratio observed for ultrasonically pretreated samples can be attributed to ultrasound-induced loosening of cellular structure and mitigation of excessive shrinkage during drying, all of which facilitate water absorption [17]. Furthermore, hot water blanching further improved the performance of ultrasound pretreatment by softening the cell wall matrix and improving energy transmission, thereby promoting more efficient moisture uptake and resulting in a higher rehydration ratio for US30B compared with the other treatments.

Furthermore, rehydration efficiency is strongly governed by the preservation of capillary pathways and cellular integrity during drying [29]. Accordingly, the higher structural stability of hydrothermally treated holy basil, associated with pectin gelatinization, restricted pore expansion and reduced water ingress during rehydration compared with ultrasonically treated samples, even though this structural stabilization was advantageous for shrinkage prevention. In contrast, the higher rehydration ratio observed for US30B at the effective rehydration time is consistent with its reduced shrinkage and high D_eff_, indicating that enhanced moisture transport combined with moderated structural deformation during drying preserved continuous pathways for water uptake during rehydration.

### 3.7 Color characteristics of dried holy basil leaves under different pretreatments

Pretreatment resulted in significant variations in the color characteristics of dried holy basil leaves, as evidenced by Table 5 (p ≤ 0.05). US30B exhibited the most negative a* values and the highest L* values, indicating superior green color retention and a lighter appearance compared with ultrasound-only treatments and the control. These color attributes were accompanied by the lowest ΔE, reflecting reduced overall color degradation during drying. In contrast, the control and ultrasound-only treatments showed significantly higher a* and ΔE values, indicating greater chlorophyll degradation and browning. This behavior can be attributed to prolonged drying time under these treatments, which likely accelerated enzymatic oxidation reactions [30]. In addition, at lower moisture levels, Maillard reactions may have occurred, contributing to increased browning of dried holy basil leaves [17]. For hydrothermally treated samples, effective inactivation of peroxidase enzymes during pretreatment contributed to improved color preservation relative to ultrasound-only and control samples [30]. However, the significantly lower L* values observed after blanching may be attributed to starch gelatinization, vitamin C oxidation, and caramelization, as reported for dried white cabbage and potato [29], [38]. In contrast, the higher L* values observed under ultrasonic treatment may be associated with hydrogen peroxide formation during sonication, which can delay browning reactions [39]. Therefore, by mitigating the individual drawbacks of blanching and ultrasonication, US30B exhibited superior color stability, likely due to the complementary effects of blanching and ultrasonication. This behavior is consistent with its lower shrinkage, shorter drying time, higher D_eff_, and improved structural integrity, which together may be related to reduced exposure to degradative conditions during dehydration. Although SB also exhibited relatively low ΔE values, its color retention remained slightly inferior to that of US30B, suggesting that the combined ultrasonic–hydrothermal pretreatment provided more effective stabilization of tissue structure and pigments during drying.

Interestingly, after 25 min of rehydration, dried holy basil leaves subjected to US30B maintained significantly greater color stability than the other samples, as shown in Table 6. Under this condition, US30B exhibited color values closest to those of the fresh leaf, characterized by a relatively high L* and a strongly negative a*. This behavior was also accompanied by the lowest ΔE, suggesting minimal irreversible color degradation during the drying–rehydration cycle. In contrast, US30 and US60 showed markedly less negative a* values and the highest ΔE. The inferior color recovery of the control further highlights the importance of pretreatment in preserving pigment stability and tissue integrity. The superior color restoration observed for US30B is consistent with its moderated shrinkage and enhanced rehydration behavior, indicating improved preservation of pigment-containing cellular structures during drying. This structural preservation contributed to a lower ΔE, reflecting reduced irreversible color change throughout the drying–rehydration process. From a practical perspective, lower ΔE values are generally associated with higher consumer visual acceptance, suggesting that US30B may better meet quality expectations for dried holy basil products.

**Table 6:** Color values of rehydrated holy basil leaves (25 min of rehydration).

Pretreatment	L*	a*	b*	ΔE
Fresh leaf	35.78 ± 0.13^a^	–9.45 ± 0.11^a^	21.02 ± 0.08^a^	−
US30	34.62 ± 0.11^d^	–3.48 ± 0.13^e^	14.29 ± 0.10^c^	9.08 ± 0.03^a^
US60	34.87 ± 0.11^c^	–3.51 ± 0.11^e^	14.19 ± 0.09^c^	9.10 ± 0.02^a^
US30B	35.24 ± 0.11^b^	–8.28 ± 0.10^b^	13.25 ± 0.10^e^	7.88 ± 0.02^e^
B	31.73 ± 0.09^f^	–8.06 ± 0.07^c^	14.10 ± 0.18^c^	8.15 ± 0.14^d^
SB	31.64 ± 0.10^f^	–7.62 ± 0.12^d^	13.45 ± 0.10^d^	8.83 ± 007^b^
C	32.56 ± 0.12^e^	–2.28 ± 0.10^f^	17.68 ± 0.12^b^	8.54 ± 0.04^c^

Different letters in each column indicate significant differences at p ≤ 0.05.

### 3.8 Chlorophyll content in dried holy basil leaves under different pretreatments

Chlorophyll is important in dried holy basil because it governs green color retention, which strongly influences visual quality, perceived freshness, and consumer acceptance. As shown in Table 7, pretreatment significantly affected chlorophyll *a* and chlorophyll *b* contents in dried holy basil leaves (p ≤ 0.05). US30B exhibited the highest retention of both chlorophyll *a* (8.884 mg/g) and chlorophyll *b* (13.184 mg/g), followed by ultrasonic and hydrothermal pretreatments, whereas the control sample exhibited the lowest chlorophyll *a* (3.853 mg/g) and chlorophyll *b* (5.902 mg/g). Furthermore, US30 and US60 retained significantly higher chlorophyll contents than hydrothermal treatments. This behavior may be associated with ultrasound-induced changes in cellular membrane permeability reported in plant tissues, which facilitates interactions between enzymes and chlorophyll precursor compounds, as reported for dried daylilies [40]. While Sledz, et al. [27] reported that prolonged drying time impairs chlorophyll molecules, the present results indicate that pretreatment effects exerted a stronger influence on chlorophyll retention than drying time alone. Accordingly, although hydrothermal pretreatment reduced drying time and increased D_eff_, its ability to preserve chlorophyll was inferior to ultrasonication, suggesting that thermal exposure during pretreatment outweighed the benefits of accelerated moisture removal.

**Table 7:** Chlorophyll *a* and Chlorophyll *b* contents in dried holy basil leaves subjected to different pretreatments.

Pretreatment	Chlorophyll Content (mg/g)	
	Chlorophyll *a*	Chlorophyll *b*
US30	7.270 ± 0.003^c^	11.631 ± 0.004^c^
US60	7.916 ± 0.006^b^	12.377 ± 0.006^b^
US30B	8.884 ± 0.008^a^	13.184 ± 0.008^a^
B	4.688 ± 0.007^d^	7.960 ± 0.002^d^
SB	4.644 ± 0.021^e^	7.689 ± 0.008^e^
Control	3.853 ± 0.012^f^	5.902 ± 0.001^f^

Different letters within each column indicate significant differences at the 0.05 level.

The markedly lower chlorophyll contents observed in B and SB treatments reflect the high susceptibility of chlorophyll to thermal degradation and pheophytinization, as high-temperature blanching promotes pigment demetallation and leaching [41]. Similar behavior has been reported in cashew apple, where blanching caused greater nutrient losses than ultrasound pretreatment, while ultrasound preserved phenolics and minimized color changes [37]. In contrast, the combined US30B pretreatment effectively balanced enzyme inactivation with reduced thermal stress and shortened drying time, resulting in more efficient preservation of chlorophyll structure than hydrothermal treatment alone. The higher chlorophyll retention in US30B corresponded to more negative a* values and lower ΔE, indicating improved green color intensity and reduced overall color change after drying (Table 5). A similar improvement has been reported in dried onions, where ultrasound pretreatment improved color retention and protected heat-labile bioactive relative to blanching alone [42].

### 3.9 Antioxidant capacity and bioactive compounds in dried holy basil leaves under different pretreatments

Antioxidant activity (DPPH), total phenolic content (TPC), and total flavonoid content (TFC) were determined only for US30B, B, and the control samples. This selection was based on a process-oriented screening strategy. US30B exhibited the best overall performance in terms of drying kinetics, structural preservation, color stability, and energy efficiency, and was therefore identified as the optimized treatment. Hot water blanching (B) was included due to its practical industrial applicability and relatively good energy performance. The control sample served as a baseline reference to evaluate the net effect of pretreatment. Treatments showing inferior process and physical performance were excluded from detailed biochemical analysis to focus on the most promising and industrially relevant conditions. As shown in Table 8, pretreatment significantly influenced DPPH, TPC, and TFC of dried holy basil leaves (p ≤ 0.05). The control sample exhibited the lowest DPPH activity as well as the lowest phenolic and flavonoid contents. This result highlights the importance of applying pretreatment prior to hot air drying. As expected, US30B exhibited the highest DPPH activity. TPC and TFC were also highest in US30B. These results reflect improved preservation of antioxidant, phenolic, and flavonoid compounds during drying. In contrast, B exhibited intermediate values for all bioactive parameters.

**Table 8:** Antioxidant activity (DPPH), total phenolic content, and total flavonoid content of dried holy basil leaves subjected to different pretreatments.

Pretreatment	DPPH (mg trolox equivalents/g sample)	TPC (mg GAE/g sample)	TFC (mg catechin/g sample)
US30B	3.789 ± 0.062^a^	5.442 ± 0.001^a^	6.826 ± 0.110^a^
B	3.331 ± 0.031^b^	4.202 ± 0.014^b^	5.066 ± 0.023^b^
Control	3.382 ± 0.057^b^	4.097 ± 0.014^c^	4.446 ± 0.010^c^

The superior retention of antioxidant and bioactive compounds observed for US30B is associated with its shorter drying time and higher D_eff_, which reduced the duration of exposure to degradative conditions and limited flavonoid degradation. Similar behavior was reported by Xi, et al. [31] during potato slice drying, where phenolic compounds were found to be highly susceptible to oxidation and thermal degradation, and ultrasound application significantly improved total phenolic retention. The authors further noted that ultrasound-assisted treatments enhanced protective effects on phenolics by improving mass transfer and reducing oxidative stress. Consistent results were also reported by Tekin and Baslar [43] for ultrasound-assisted vacuum drying of red pepper, where ultrasound application promoted higher retention of bioactive compounds. The subsequent application of blanching following ultrasonication further beneficially modified tissue structure and shortened drying duration. Accordingly, antioxidant and bioactive compound retention followed the order US30B > B > control. Furthermore, the higher antioxidant and bioactive retention observed for US30B is consistent with its improved green color (more negative a*) and higher chlorophyll retention. This suggests that preservation of photosynthetic pigments was accompanied by reduced oxidative degradation of bioactive compounds.

### 3.10 Multivariate analysis of drying performance and quality attributes

**Fig. 7:**
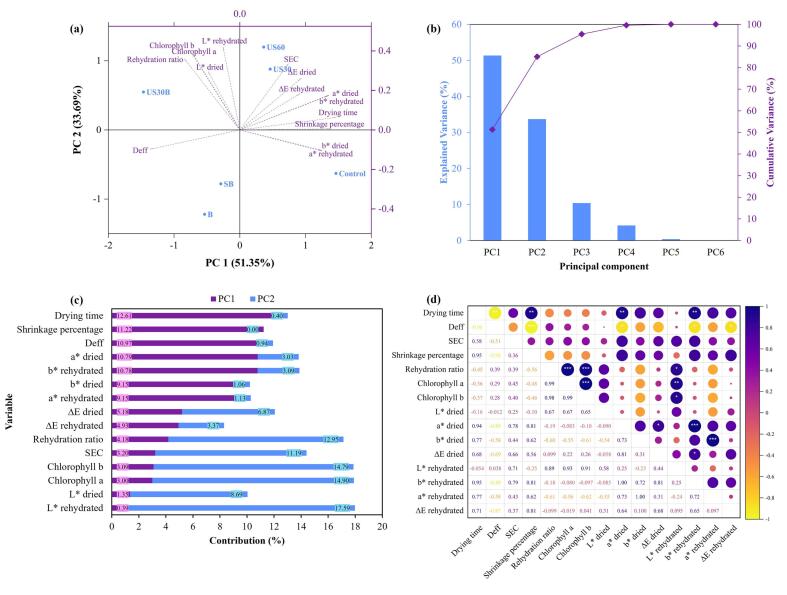
Principal component analysis of sample quality attributes: (a) PCA score and loading biplot illustrating sample distribution and variable correlations; (b) explained and cumulative variance of principal components; (c) contribution of each variable to PC1 and PC2; and (d) correlation matrix of the measured quality attributes (p < 0.05, p < 0.01, p < 0.001).

Fig. 7 illustrates the principal component analysis (PCA) and Pearson correlation of the measured drying performance and quality attributes. The variability of drying-related quality attributes can be explained by five principal components, with PC1 accounting for 51.35% of the total variance and capturing most of the variability. This PC1 was strongly associated with drying time and shrinkage percentage, both of which exhibited high positive loadings along PC1 and showed high contributions of 12.61% and 11.22%, respectively. The positive correlation (r = 0.95, p < 0.01) between drying time and shrinkage percentage along PC1 indicates that prolonged drying was associated with increased structural deformation of the holy basil tissue. This observation is consistent with previous studies reporting cell wall collapse and volumetric contraction during moisture removal in biological materials [33], [44].

Conversely, the effective moisture diffusivity (D_eff_) showed a strong negative loading along PC1, indicating an inverse relationship with drying-induced changes. This suggests that higher D_eff_ values were associated with reduced shrinkage and shorter drying time. Previous studies have reported that enhanced moisture diffusivity can improve the retention of physicochemical properties during drying. For example, drying techniques that promote internal moisture transport, such as vacuum freeze-drying (VFD) and electrohydrodynamic drying (EHD), have been found to better preserve color, texture, and bioactive compounds compared to conventional drying methods [45], [46]. Similarly, color degradation parameters, including a^⁎^, b^⁎^, and ΔE of both dried and rehydrated leaves, were clustered in the positive PC1 region, suggesting their association with prolonged drying exposure. The grouping of these chromatic indicators alongside drying time and shrinkage reinforces the notion that pigment destabilization and color degradation were associated with prolonged thermal exposure during drying [47].

PC2 accounted for an additional 33.69% of the variance, with most variables showing positive loadings along this component, except for D_eff_, b* of dried leaves, and a* of rehydrated leaves. Among these variables, L* of the rehydrated leaves, chlorophyll *a*, and chlorophyll *b* were the primary contributors to PC2, with contributions of 17.59%, 14.90%, and 14.79%, respectively. The high positive loadings of these variables along PC2 suggest that their variability is distinct from that of D_eff_. Similar findings have been reported in ultrasound-assisted drying studies, where pigment stability was shown to depend more strongly on internal mass transfer enhancement rather than solely on drying intensity [48]. Notably, the rehydration ratio exhibited a positive association with chlorophyll retention variables along PC2, indicating that pigment preservation contributes to improved post-drying reconstitution behavior. This relationship suggests that reduced thermal damage to cellular membranes enhances the capacity of dried materials to reabsorb water during rehydration, thereby improving functional quality.

Samples pretreated with ultrasonic-assisted blanching (US30B) were positioned closer to the PC2-positive region, reflecting improved preservation of rehydration ratio, chlorophyll content, and L*. This indicates that the combined ultrasonic and blanching pretreatment enhanced moisture removal efficiency, resulting in a shorter drying time under the same hot air drying temperature. In contrast, control samples were predominantly distributed along the positive PC1 axis, indicating greater susceptibility to drying-induced deterioration manifested by increased shrinkage and color deviation. The correlation matrix further confirmed strong positive relationships between drying time, SEC, and shrinkage percentage (p < 0.01), suggesting that prolonged and energy-intensive drying conditions are associated with greater structural degradation. Based on the PCA distribution, US30B demonstrated a favorable balance between structural integrity and pigment retention compared to other treatments. Therefore, US30B can be considered the most effective pretreatment condition for preserving both physicochemical and optical quality attributes during hot air drying.

Similar findings were reported for mango peel drying, where pretreatment improved drying efficiency and lowered energy demand, supporting its relevance for industrial applications [49]. From an industrial perspective, such pretreatments can decrease specific energy consumption and drying time, thereby improving overall economic feasibility. However, advanced preprocessing techniques such as ultrasonication may involve higher capital investment, necessitating a careful balance between energy savings and equipment costs. In this context, the use of conventional hot air drying can partially offset these costs, as it requires lower capital investment compared to advanced drying technologies such as microwave-assisted systems [50]. Overall, the superior performance of US30B confirms that combined pretreatment effectively enhances drying performance and quality of dried holy basil, making it a promising strategy for efficient and scalable drying processes. Nevertheless, a comprehensive techno-economic analysis is still required to fully evaluate its industrial feasibility.

## 4 Conclusions

This study shows that the hot air-drying performance of holy basil can be substantially enhanced through the combined application of ultrasonic and hydrothermal pretreatments. The integrated pretreatment increased effective moisture diffusivity by threefold, shortened drying time from 100 to 30 min, and reduced specific energy consumption by 11%. These improvements in drying kinetics were accompanied by minimized shrinkage, enhanced rehydration capacity, and superior preservation of quality attributes, including green color, chlorophyll, phenolic compounds, flavonoids, and antioxidant activity. The results indicate that enhancing internal moisture transport during pretreatment may be more effective than simply increasing drying temperature for improving drying efficiency and product quality.

## CRediT authorship contribution statement

**Suluh Pambudi:** Writing – review & editing, Writing – original draft, Visualization, Methodology, Investigation, Formal analysis, Data curation, Conceptualization. **Duanghathai Khamon:** Methodology, Investigation, Formal analysis, Data curation, Conceptualization. **Tai Van Ngo:** Formal analysis, Data curation. **Wanphut Saechua:** Writing – review & editing, Conceptualization. **Jiraporn Sripinyowanich Jongyingcharoen:** Writing – review & editing, Validation, Supervision, Resources, Project administration, Methodology, Funding acquisition, Conceptualization.

## Declaration of competing interest

The authors declare that they have no known competing financial interests or personal relationships that could have appeared to influence the work reported in this paper.
